# Analysis of a Cementless Femoral Stem Neck Fracture Using Scanning Electron Microscopy and the Finite Element Method

**DOI:** 10.1155/2019/7204598

**Published:** 2019-05-07

**Authors:** Hirokazu Takai, Daisuke Nakayama, Masatoshi Murayama, Tomoki Takahashi

**Affiliations:** Department of Orthopaedic Surgery, Kumamoto Kinoh Hospital, Kumamoto, Japan

## Abstract

Implant fracture is one of the rarest complications of total hip arthroplasty (THA). A 57-year-old woman experienced a fracture of the femoral stem (AHFIX Q, KYOCERA, Japan) about five years after THA. We examined the broken stem by digital microscopy, scanning electron microscopy, and finite element method. The anterolateral corner of the stem's neck was found to be the origin point of the fracture. Finite element method analysis revealed that the stress concentration was highest in the corner of the hollow for apparatus attachment. The stem's design has been considered one of the risk factors for stem fracture. In this patient, multiple risk factors, including thin stem (the smallest size, NAR #1), use of the long neck (+3 mm), obesity (body mass index: 27.3), and adjacent osteoarthritis (contralateral THA loosening and knee osteoarthritis), were present. To our knowledge, this is the first reported case of an AHFIX Q stem fracture. Surgeons must keep in mind that fracture of the femoral stem in patients with several risk factors is possible even several years after THA.

## 1. Introduction

Implant fracture is one of the rarest complications of total hip arthroplasty (THA), with an estimated prevalence of 0.27% after THA [1]. Multiple articles about femoral stem fractures after THA have previously been published [2–7]. However, no cases of AHFIX Q stem (KYOCERA, Japan) fractures have been reported, although approximately 30000 isomorphic stems have been used since 1999. We present the case of a 57-year-old woman who experienced a cementless femoral stem fracture about five years after THA. This is the first case report of an AHFIX Q stem fracture. The aim of this case report was to alert for the risk of femoral neck fracture due to the design of this specific implant and to investigate other associated factors.

The patient was informed that all data concerning this case would be submitted for publication, and she provided informed consent for publication. The study design was approved by the appropriate ethics review board.

## 2. Case Presentation

The AHFIX Q stem is an uncemented, proximally fixed, fit and fill-type stem made of a Ti-6Al-2Nb-1Ta-0.8Mo alloy. The proximal part of the stem was heat-treated with alkali to obtain bone implant fixation [8–10]. AHFIX Q stems are of either standard proximal size (STD) or narrow proximal size (NAR), with or without a collar. The stem has a deep hollow in its shoulder for apparatus attachment and a 9-10 mm neck taper.

**Figure 1:**
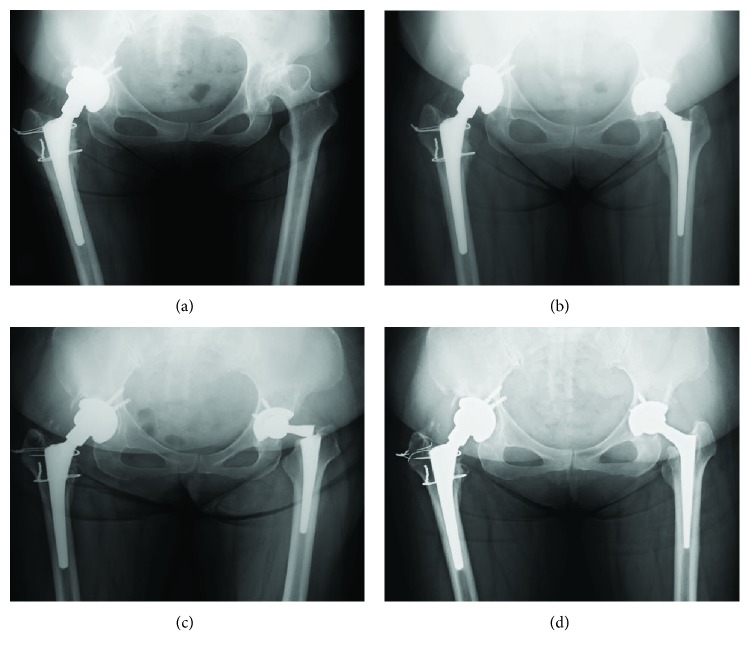
Radiographs. (a) Preoperative radiograph showing severe osteoarthritis of the left hip. (b) Postoperative radiograph of primary THA of the left hip using AHFIX Q stem (NAR #1). (c) Radiograph showing breakage of the AHFIX Q stem at the base of the neck. (d) Postoperative radiograph after revision THA of the left hip. THA: total hip arthroplasty.

A 57-year-old woman (body weight: 70 kg, body mass index: 27.3 kg/m^2^) had severe bilateral coxalgia. She had undergone right THA at another hospital. Because of early aseptic loosening and recurrent dislocation of the hip, four revision hip arthroplasties had been performed within one year after the first right THA. After these interventions, she presented at our hospital because of left coxalgia. Radiography revealed end-stage severe left hip arthritis and malposition of the cup implant on the right side (Figure 1(a)).

Left THA was therefore performed via a posterior approach using an uncemented hip stem (AHFIX Q, KYOCERA, Japan) (Figure 1(b)). The size of the AHFIX Q stem was NAR #1, the smallest size available. The stem was implanted in a neutral position with 25° of stem anteversion. A cementless acetabular cup (AHFIX Q3 shell, KYOCERA, Japan) was fixed with two screws. The acetabular cup anteversion and inclination were 15° and 42°, respectively. A 26 mm Co-Cr-Mo head with a +3 mm neck was installed with high cross-linked polyethylene liner. The University of California at Los Angeles (UCLA) activity score improved from 3 to 6. She was discharged from our hospital after complete recovery from left coxalgia shortly thereafter. Four years and eight months after left THA, she developed severe left thigh pain without any trauma. Radiography revealed an uncemented femoral stem fracture at the base of the neck (Figure 1(c)). Revision THA was immediately performed using a Wagner cone stem (Zimmer Biomet, Warsaw, IN, USA, Figure 1(d)). At the final follow-up visit, although she felt instability on her right hip, she managed to walk without coxalgia. We proposed revision THA on the right side several times; however, the operation has been delayed because of the patient's health condition. There has been no early loosening of the left prosthesis until now.

**Figure 2:**
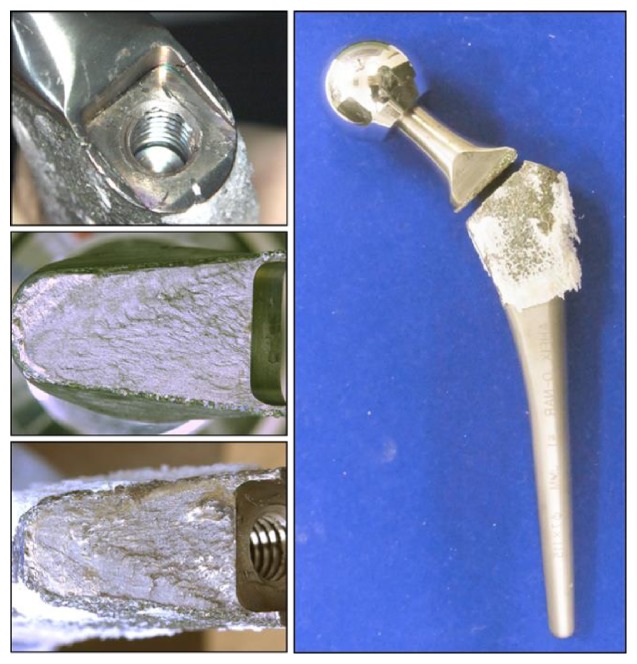
Appearance of the broken stem. Macroscopic appearance of the broken AHFIX Q stem at the base of the neck. The proximal coating area shows good bone ingrowth. The base of the neck has a sharp slot for handle attachment and the corner is not rounded but is angular.

**Figure 3:**
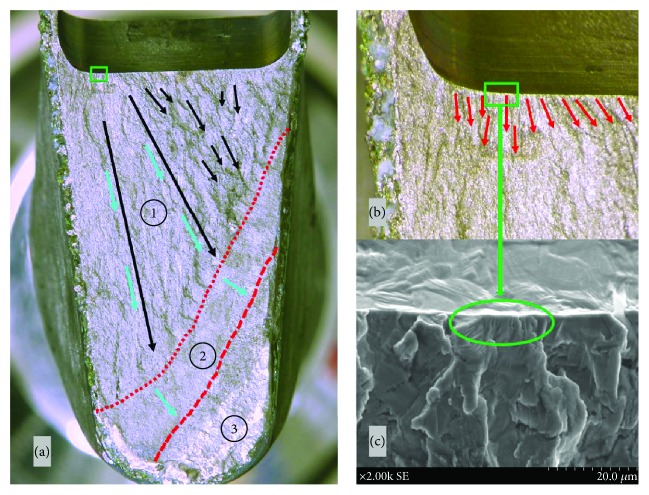
Fractured surface observed by macroscopy, digital microscopy, and SEM. (a) Fracture surface observed macroscopically. Arrows indicate the advancing direction of the progressive destruction. (b) Fracture surface observed by digital microscopy. There were multiple micro streaks from the edge of the attachment slot in the stem corner (red arrows). (c) Picture of the fractured surface observed by scanning electron microscopy (~×2000). The basis of the streaks was assumed to be the origin of the fracture. SEM: scanning electron microscope.

**Figure 4:**
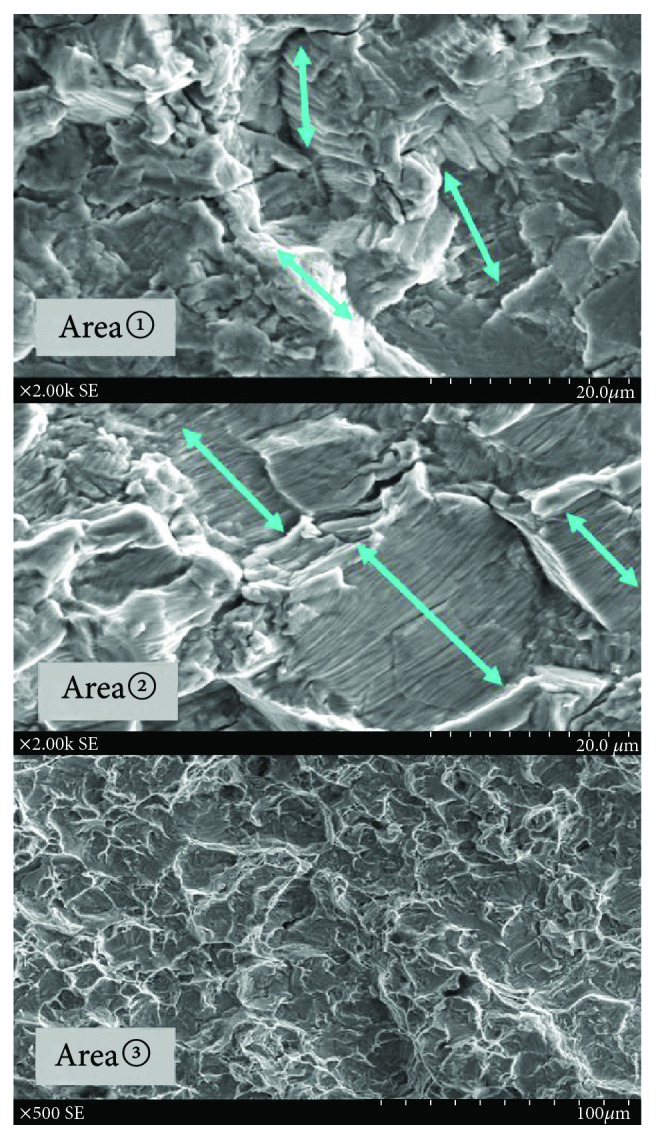
Analysis by SEM. The SEM images show many parallel striations in area 1 and area 2. Furthermore, there are many dimples in the area 3. SEM: scanning electron microscope.

To find out the cause of fracture, we performed various analyses. The surface of the broken stem was observed under a digital microscope (~×40, VHX-200, KEYENCE, Japan) and scanning electron microscope (SEM; ~×2000, S-3400N, HITACHI, Japan). The proximal coating area of the stem showed good bone ingrowth macroscopically (Figure 2). Observation under a digital microscope and SEM showed some cracks from the anterolateral edge of the fracture surface at the shoulder corner of the stem's neck, which had a hollow junction for apparatus attachment. The cracks extended from the anterolateral surface to the posteromedial surface (Figure 3). The fracture surface was divided into three areas according to the form. There were multiple micro streaks from the anterolateral edge to the medial side, accompanied by a wavy undulation in area 1. Based on these findings, the anterolateral edge was assumed to be the origin of the fracture. The surface in area 2 was flatter than that in area 1. This difference showed that the fracture reached the posterior wall and that the fracture progress had changed. There were multiple striations in areas 1 and 2 (Figures 3 and 4). Striations showed a typical striped pattern with parallel streaks formed by repeated enlargement and destruction of the material caused by repetitive loading and usually appear in fatigue fractures. Furthermore, there were multiple dimples in area 3 (Figures 3 and 4). Dimples are dent-connecting microcavities caused by local tissue rupture, which typically appear in static fractures. Our findings suggested that the anterolateral corner of the stem's neck was the starting point of the fracture and that the fracture had spread from the anterolateral to the posteromedial aspect. Repeated loading led to progressive metal fatigue, which ultimately led to a static fracture.

**Figure 5:**
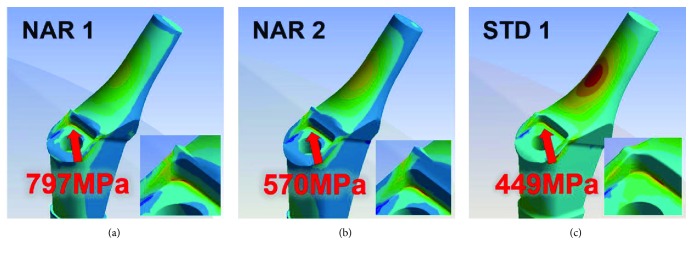
Result of the analysis by FEM. FEM images showing that the stress was maximum in the corner of the hollow junction for all sizes. FEM: finite element method.

The stress condition of the stem was evaluated by the finite element method (FEM; ANSYS Workbench Ver.13, ANSYS, Canonsburg, PA, USA). The subjects of FEM analysis were the top three narrowest and smallest sizes: NAR #1, NAR #2, and STD #1, considering the effect of size. In this series, FEM analysis was carried out under the assumption that five times the load of 80 kg body weight was applied to the center of the femoral head statically. A weight of 80 kg was set as the body weight applied on the stem. FEM analysis showed that the stress concentration was maximum in the anterolateral corner of the hollow junction for all sizes. The maximum stress was highest for NAR #1 (797 MPa). For NAR #2, the maximum stress was 570 MPa (a decrease of approximately 28%, Figure 5). For STD #1, the maximum stress was 449 MPa. NAR #1 had the highest stress concentration compared with all other sizes. Thus, the stress concentration at the anterolateral corner of the stem neck seems to be a factor associated with fracture of the AHFIX Q stem NAR #1.

## 3. Discussion

Various studies about femoral stem fractures after THA have previously been reported, and multiple risk factors for such fractures have been reported. The risk factors for femoral stem fractures are divided into three categories.

The first category involves patient-associated risk factors, such as obesity, high activity level, and adjacent osteoarthritis [2–6]. Harvie et al. reported that patients with BMI > 30 kg/m^2^ were at an especially high risk for a stem fracture [3]. The UCLA activity score of this patient improved from 3 to 6 (regularly participates in moderate activities). This is a moderate activity level in a healthy person; however, it might represent an overload in patients with adjacent osteoarthritis (contralateral THA loosening and knee osteoarthritis).

The second category of risk factors involves the surgical technique, for example, using an undersized stem or a long neck [2–6]. Malposition of the implant, use of an outdated poor cementing technique, or performing extended trochanteric osteotomy were also reported as risk factors for stem fracture [2].

The third category of risk factors involves the implants themselves. Regarding fracture of the femoral stem, many articles have reported negative results when using modular-type stems [11, 12]. A thinner stem (neck and body) can cause stress concentration. A size of 9-10 mm taper trunnion may be too thin for obese individuals. The stem production method is also considered a risk factor. Laser etching at the neck or neck-shoulder junction of the stem causes stress risers, which may lead to a fatigue fracture [13–15].

Since 1999, approximately 30000 isomorphic cementless stems have been used and have yielded good results [9]; however, no reports on fractures of AHFIX Q stem implants are available. Our patient had several risk factors for a femoral stem fracture, including a thin stem, obesity, and adjacent osteoarthritis. Among them, the stem shape, particularly the hollow space for apparatus attachment, was considered one of the main reasons for stress concentration. Repeated load onto this fragile point led to metal fatigue and subsequent fracture.

**Figure 6:**
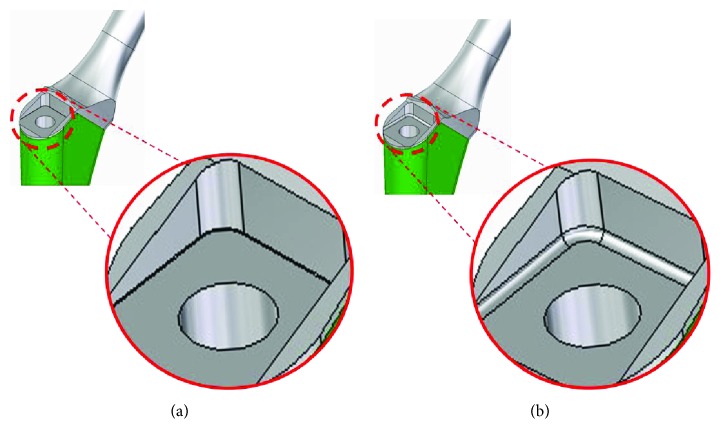
Improvement of the AHFIX Q stem design. (a) Conventional AHFIX Q stem design. (b) In the new design, the shoulder corner of the hollow had a curve (radius = 0.5 mm). This improvement of the design decreases the stress concentration.

Upon analysis, it was found that the breaking strength of the smallest size (NAR#1) is much weaker than that of the other sizes. Subsequent to this case, the shape of the hollow for stem removal has been improved. The new design of the shoulder corner of the hollow has a curve (radius = 0.5 mm, Figure 6). This improvement in the design decreases the stress concentration and increases the breaking strength. The fatigue strength of NAR #1 improved 33% from 2.0 kN to 3.0 kN, while the fatigue strength of NAR #2 improved from 2.8 kN to 5.0 kN. After the improvement of the stem, manufacturers restricted the use of the NAR #1 to patients with a body weight under 60 kg. Fractures of the AHFIX Q femoral stem have not been reported after this improvement.

This study's limitations are that the strength test was performed in vitro and the size of the head was only 22 mm (+0). At the beginning of the AHFIX Q stem developing, the use of a 22 mm head was standard practice in THA.

Surgeons must keep in mind that fracture of the femoral stem in patients with several risk factors is possible even several years after THA.
